# Influence of ZnO, SiO_2_ and TiO_2_ on the aging process of PLA fibers produced by electrospinning method

**DOI:** 10.1007/s10973-019-08890-6

**Published:** 2019-11-02

**Authors:** Karolina Kosowska, Piotr Szatkowski

**Affiliations:** Department of Biomaterials and Composites, Faculty of Materials Science and Ceramics, AGH University of Science and Technology, Al. Mickiewicza 30, 30-059 Krakow, Poland

**Keywords:** Poly(lactic acid), Aging process, Fibers, Electrospinning

## Abstract

The aim of this work was to study the effect of ceramics particles addition (SiO_2_, ZnO, TiO_2_) on the ultraviolet (UV) aging of poly(lactic acid) nonwovens fabricated using electrospinning method. The resistance to aging is a key factor for outdoor and medical applications (UV light sterilization). Nonwovens were placed in special chamber with UV light. Changes of physicochemical properties were recorded using differential scanning calorimetry and attenuated total reflection Fourier-transform infrared spectroscopy. The fibers’ morphology was studied by using scanning electron microscopy. Obtained results clearly showed that only PLA fibers with ZnO particles gained an increase in UV resistance. The paper presents a description of structural changes taking place under the influence of UV aging processes and describes the mechanisms of this process and the effect of ceramic addition on the lifetime of such materials.

## Introduction

Biodegradable and bio-based polymers have recently drawn more and more attention in several applications, including composites [1–3], packaging [4] and medicine [5]. Polyesters are considered to be one of the most important classes of biomaterials [6]. Poly(lactic acid) (PLA), linear polyester, is extensively tested in medicine and tissue engineering, due to its biodegradability, non-toxicity and biocompatibility [7, 8]. PLA is a thermoplastic relatively easy to process. In addition, its mechanical properties are similar to commonly used synthetic polymers. Physicochemical properties of PLA depend on a stereoisomeric form of polymer [9]. Lactic acid (LAc), a repetitive unit of PLA, consists of two optical isomers: D(−)LAc and L(+)LAc. Homochiral PLA is a semi-crystalline, isotactic polymer (PLLA and PDLA). Polymerization of D- and L-LAc mixture leads to the formation of heterochiral, atactic PLDLA with amorphous properties.

The PLA is susceptible to hydrolysis and under the conditions of the human body enzymatically decomposes into LAc, which occurs naturally in living organisms. In the citric acid cycle, LAc is converted into carbon dioxide and water [10]. Thanks to good mechanical properties that allow PLA to endure the stresses applied in a human body; it is commonly used in implants for bone fixation and absorbable surgical sutures [11].

In tissue engineering, PLA has been utilized as material for drug delivery microspheres [12, 13] and scaffolds [14, 15] for cell growth. It is required for scaffold to support regeneration of the tissue in the place of the defect and then degraded when the healing is completed. PLA could be easily made in various forms and shape, including microspheres and fibers with diameter that ranges from nanometers to micrometers. The main advantage of forming 2D and 3D fibrous scaffolds is a strong anisotropy of properties that allows to adapt material to specific requirements, a very high surface area, and the possibility of creating complex microstructures with specific pores and density [16]. Thanks to the ability to control the physicochemical properties of fibers; also through the degree of crystallinity, modifications of the volume and surface of the fibers, it is possible to modulate the biological response [17].

Here, we report the fabrication of PLA fibers modified with inorganic particles (ZnO, SiO_2_, TiO_2_) using the electrospinning method (ES). This technique involves electrostatic forces to generate polymer solution jets and induce the ejection through a spinneret. An electrical potential is applied between the tip of a needle and a grounded collector. In order for the polymer jet to be ejected, the electric field must overcome the surface tension of the drop. The fiber morphology is influenced by many parameters, especially concentration of the polymer, molecular weight, distance between tip and collector, solvent content, flow rate, applied voltage and temperature [18–20].

PLA exhibits disadvantages in some key aspects, such as hydrolysis and degradation under UV light exposure [21], what limits its applicability. The resistance to aging is a key factor for outdoor and medicine applications (packaging, stitches, bandage, sterilization). UV stability of PLA-based materials has attracted a lot of attention recently [21–23]. However, to the best of our knowledge, there are no studies in the literature regarding the influence of UV exposure on the properties of PLA nanocomposite fibers. We hypothesized that modification of fibers with inorganic particles (SiO_2_, ZnO and TiO_2_) can affect the stability of PLA. ZnO and TiO_2_ are effective absorbers of UV [24, 25] and are commonly used as UV-screen agents in sunblocks. Zhang reported that ZnO nanoparticles can stabilize poly(butylene succinate-co-butylene adipate) matrix and hinder the photodegradation of polymer [26].

## Experimental

### Materials

Poly(lactic acid) (PLA, IngeoTMBiopolymer 3251D) with molecular weight *M* = 70,000–120,000 g mol^−1^ was purchased from NatureWorks LLC, USA. Dichloromethane (DCM) and dimethylformamide (DMF) were obtained from Avantor Performance Materials Poland S.A., Gliwice, Poland. Ceramic particles: ZnO particles (1 μm), SiO_2_ nanaoparticles (12 nm) and TiO_2_ nanoparticles (100 nm) were purchased from Merck KGaA, Germany.

### Preparation of PLA-based solutions for electrospinning process

PLA-based solutions for electrospinning process (ES) with concentrations 11, 13 or 15% (w/v) were prepared by dissolving polymer in binary-solvent system of DCM and DMF (2.5:1 v/v or 3:1 v/v). Solution was stirred for 24 h using magnetic stirrer at about 50 °C. Next, ceramic particles powder (ZnO, TiO_2_ or SiO_2_) was added and solution was ultrasonicated for 10 min. Next, PLA solution was stirred for another 24 h. The concentration of inorganic particles was 6 mass%, beyond that content solution retained its rheological properties.

### Electrospinning process

PLA-based nonwoven was formulated through electrospinning process (ES) using apparatus constructed at Department of Biomaterials and Composites, AGH, Poland. The solution was sonicated for 10 min before injection into 10 mL syringe. A high voltage–power supply (0–25 kV) was used to generate an electric field between needle (0.7 mm) and cylindrical, rotating collector (width: 5 cm) covered with aluminum foil. The system was encased in a special chamber that allowed to perform ES process at constant temperature (50 °C) and humidity (10%). The spinning time of nonwovens was 1 h.

### Aging test

**Fig. 1:**
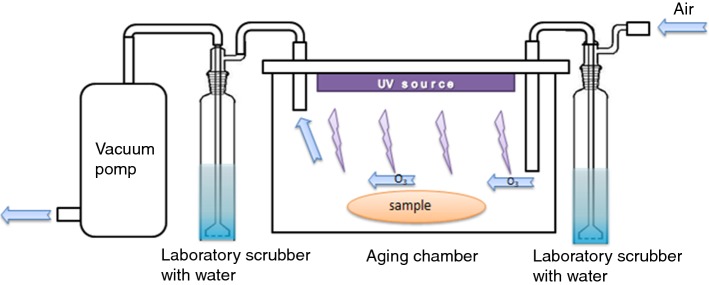
The chamber for UV aging test, equipped with UV-lamp and system of distilled water vessel, pump and piping system forcing air circulation

The aging test was carried out by exposing PLA and PLA-composite nonwovens to UV light. Samples were placed into the chamber equipped with UV-C lamp (35 W cm^−2^). Air circulation was forced in the chamber (Fig. 1) to remove ozone, which was forming from oxygen by UV irradiation during aging test. The presence of ozone could accelerate degradation of PLA nonwoven. The equivalent of exposure time to UV radiation was calculated from the average exposure the Earth to Sun’s UV radiation (62 W m^−2^ [27, 28]). One hour in the aging chamber equals 5645 h of exposure to solar radiation, assuming that the Sun shines continuously and all UV irradiation reaches the Earth’s surface. The samples were exposed to UV light for 15 min, 1 and 4 h.

### Characterization

Surface morphology of PLA nonwovens and composites fibers was studied using scanning electron microscope (SEM, Nova NanoSEM 200) with an accelerating voltage of 18 kV. Samples were mounted onto special holders and coated with conductive carbon layer prior to SEM analysis. Images analysis software (ImageJ) was applied to determine fiber’s diameters and distribution of fibers’ directions in nonwovens. For each material, 30 fibers were measured. Differential scanning calorimetry (DSC) measurements were taken using DSC1 (Mettler Toledo) in dynamic mode (atmosphere: nitrogen, 200 mL min^−1^, heating and cooling rates: 10 K min^−1^, temperature range: − 30–180 °C). Samples (6 mg) were sealed in aluminum pans and placed in the equipment sample chamber. Fourier-transform infrared spectra (ATR-FTIR) of PLA and composite nonwovens before and after different times of UV light aging were recorded using Tensor 27 equipment with ATR mode (diamond crystal), in range 4000–600 cm^−1^, at resolution 4 cm^−1^. For each sample, 64 scans were performed.

## Results and discussion

### Optimization of electrospinning parameters

**Fig. 2:**
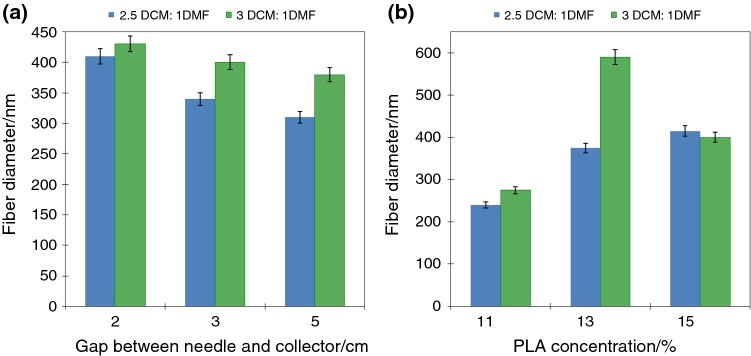
Average diameter of PLA fiber fabricated using: **a** different gap between needle and collector, **b** PLA concentration

Diameter, quality and direction of fiber arrangement can be controlled by parameters such as polymer concentration in solution, solvents, electrical field voltage, or collector rotation speed. The needle-collector gap influences jet flying time and evaporation of the solvents. Binary-solvent system (DCM and DMF) was used to fabricate PLA-based nonwovens. The SEM images were used to evaluate the quality (morphology and diameter) of obtained PLA fibers. All of the fibers had smooth and defect-free morphology. It was observed that ratio of solvent had a great influence on the fiber diameter (Fig. 2). Thinner fibers were obtained using ratio 2.5:1, regardless of the polymer concentrations. The other optimized parameter was gap between tip of needle and collector, and polymer concentration. As expected, the diameter of the fibers decreased with increasing needle-collector distance, as shown in Fig. 2a. This was due to the longer time that the solvents had to evaporate. Also, in most cases, increasing the polymer concentration resulted in obtaining fibers with increased diameters (Fig. 2b). Based on the SEM images, the following parameters were selected to obtain fibers modified with ceramic particles: concentrations of PLA—13%, gap between tip of needle and collector—5 cm, ratio of DCM and DMF—2.5 to 1.

### Impact of UV aging on fibers morphology

**Fig. 3:**
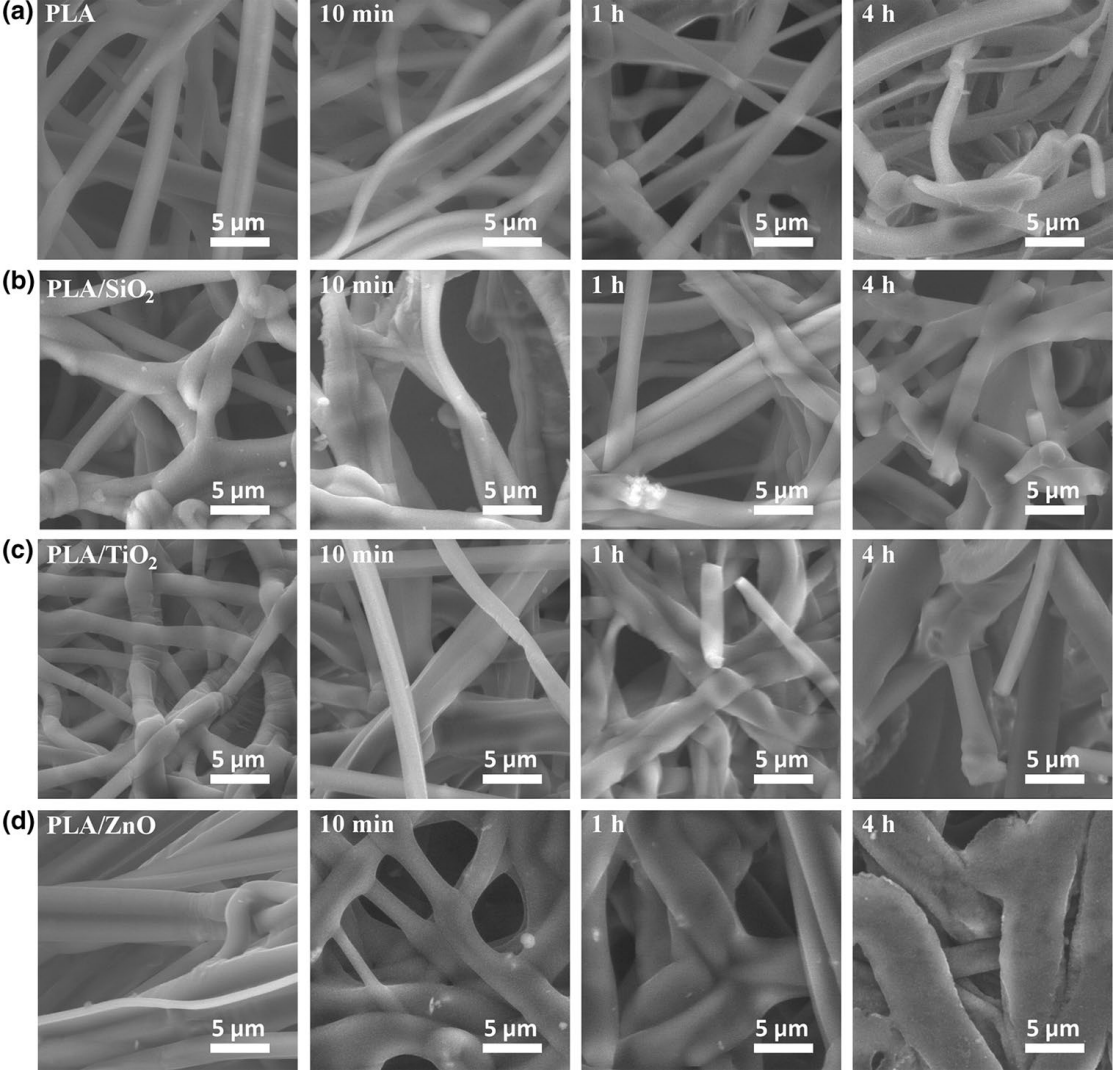
SEM images of PLA-based nonwovens before and after UV aging test (10 min, 1 h and 4 h: **a** pure PLA, **b** PLA/SiO_2_, **c** PLA/TiO_2_, **d** PLA/ZnO

**Fig. 4:**
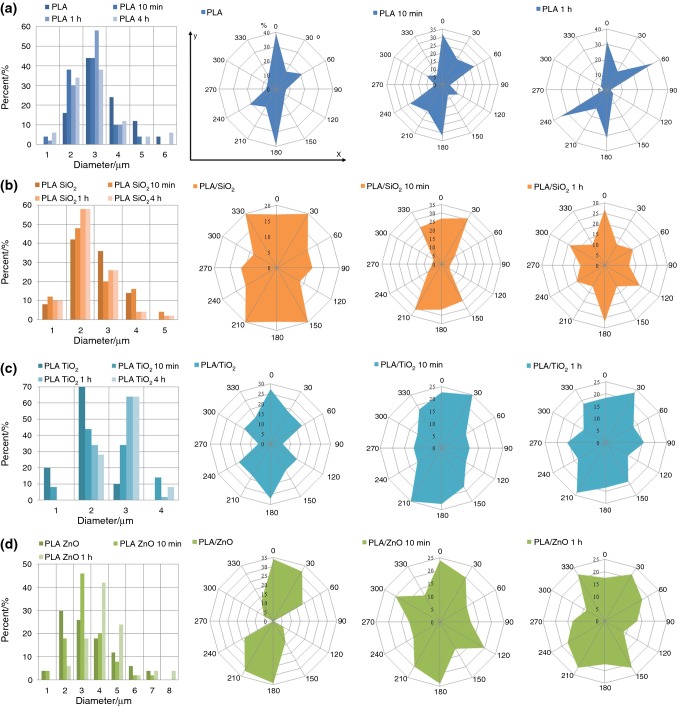
Fiber diameter distribution and fiber alignment distribution plots of: **a** PLA, **b** PLA/SiO_2_, **c** PLA/TiO_2_, **d** PLA/ZnO fibers after different times of UV exposure. *Y*-axis indicates the direction of the collector’s rotation

The morphology of the composite fiber was examined using SEM (Fig. 3). The pure PLA fibers exhibited smooth and defect-free surface, as shown in Fig. 3a. However, diameter distribution was wide, from 1.5 to 5.5 μm, with average diameter 2.91 μm (Fig. 4a). It was found that ceramic additives caused a reduction in average fiber diameter. For SiO_2_, TiO_2_ and ZnO, it was 2.12, 1.40 and 2.79 μm, respectively, as well as narrowing the distribution (Fig. 4b–d). Figure 3b, c shows that adding SiO_2_ and TiO_2_, in contrast to ZnO, changed the morphology significantly. In both cases, the fiber surface was wrinkled and defected.

The morphology of fibers after UV degradation was evaluated as well. Exposure of PLA fibers to irradiation for 10 min and 1 h did not significantly affect their morphology. After 4 h, broken and bent fibers were observed (Fig. 3a). A similar phenomenon occurred in composites with SiO_2_ and TiO_2_ (Fig. 3b, c). The PLA/ZnO fibers behaved differently under the UV exposure. Changes in morphology were much more significant. Fibers fused together and the original microstructure was completely destroyed after 4 h, as shown in Fig. 4d. In addition, the change in the distribution of the fiber alignment was also the most significant (Fig. 4d). Before the degradation, fibers were arranged almost parallel to each other, but after degradation crossed fibers appeared (Fig. 3d). However, broken fibers weren’t observed even after 4 h of UV exposure.

Fibers in PLA nonwoven were arranged parallel to the direction of the collector rotation in major part, as shown in Fig. 4a (*y*-axis). The aging process resulted with a slight change, the chart became a little wider, but in the range not exceeding 5% after 10 min of UV exposure. With longer aging time, the fibers broke during relaxing and changed the orientation, which is clearly visible on the graph for 4 h of UV exposure. This phenomenon was intensified even more in the case of composite fibers modified with TiO_2_ and SiO_2_ (Fig. 4b, c). Addition of ceramic particles did not promote receiving oriented nonwovens. They generate additional stresses in the fiber. When fiber broke under the UV radiation, it relaxed and changed its direction to a more favorable energetically. This effect is the most significant for PLA/SiO_2_ nonwoven.

The presence of ceramic particles led to larger deviations in the orientation of the nonwovens. This effect was most significant in the case of fibers with the addition of SiO_2_ particles. The spectrum of distribution of fibers collected on the collector during the ES process was the widest. The aging process also evidently led to a change in the direction of the fiber.

### Impact of UV aging on fibers thermal properties

**Fig. 5:**
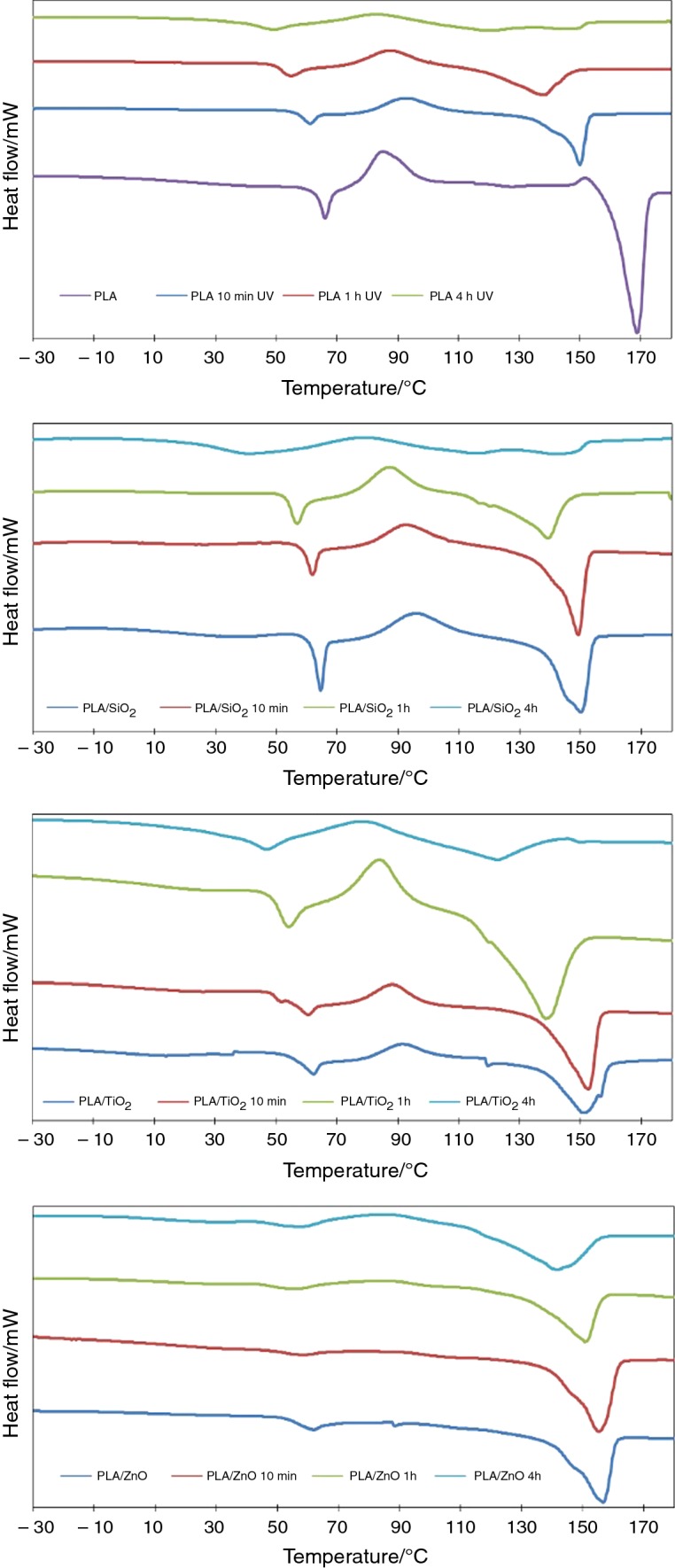
DSC curves of PLA-based nonwovens before and after UV aging: **a** PLA, **b** PLA/SiO_2_, **c** PLA/TiO_2_, **d** PLA/ZnO

Figure 5 shows DSC curves of pure PLA fibers and fibers with ceramic particles addition. PLA is a crystalline polymer. For pure PLA, three phase transitions were clearly visible on the DSC curve (Fig. 5a): glass transition with recrystallization (63.0–69.3 °C), cold recrystallization (77.0–94.4 °C), recrystallization before melting and melting (159.1–172.5 °C). These transformations were very clear, followed by each other at certain intervals of temperature. This demonstrated that well-oriented and structurally homogeneous fibers formed the nonwoven layer.

The UV degradation induced structural changes in polymer, what is directly translated into thermal properties of PLA, which can be seen in Fig. 5a. The energy of phase transformations decreased with the aging time. A decrease in glass transition temperature (*T*_g_) is due to the less need for fiber to go into a highly elastic state.

**Fig. 6:**
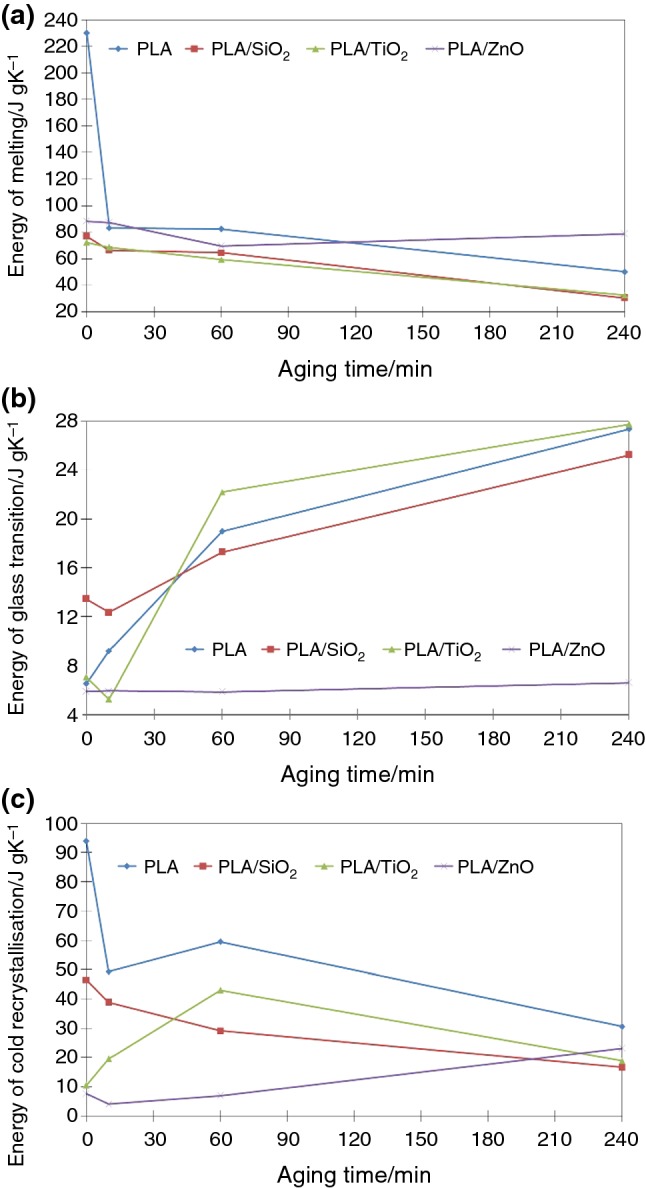
Changes of melting, glass transition and cold recrystallization energy during UV aging time

Ceramic’s additions have a significant impact on the degradation process under the UV radiation (Fig. 5b–d). It seems that TiO_2_ even accelerated the degradation of polymer structure. Addition of ceramic’s particles significantly reduced the temperatures of transformation processes and their energy. After 4 h in aging chamber, the transformations were almost invisible on the curve. Only ten degree separated melting from glass transition with recrystallization. Energy of melting decreased as the sample holding time in aging chamber increased. Similar situation was observed for fibers modified with SiO_2_. The exception here is ZnO; in this case, the drop is insignificant. The drop of melting energy after 4 h wasn’t that significant. This is consistent with SEM images; PLA/ZnO fibers were the least degraded. The exact values of phase transitions’ energies are shown in Fig. 6.

Significant differences were also noted in the case of recrystallization energy. Over time, the recrystallization energy decreases, with the exception of PLA/ZnO nonwoven. In this case, a slight increase in energy was observed. After 4 h, the energy value doubled. The opposite trend was noted for the composite fibers modified with TiO_2_ particles. After the initial increase, the transformation energy dropped significantly.

Melting of PLA/ZnO composite began at a slightly higher temperature compared to pure PLA and other composites. The energy also remained constant throughout the aging test. This can be explained by the greater proportion of the crystalline phase in the polymer. For this reason, no cold recrystallization was observed. ZnO nanoparticles may have acted as place of nucleation starting and promoted the crystallization of PLA.

The positive impact of ZnO particles on PLA stability was also demonstrated by the lack of a significant increase in glass transition energy. In fact, the value remains almost the same for 4 h of degradation. This may indicate improved stability of the composite fibers. In other cases, including pure PLA, the energy value increases several times. The most dynamic changes were observed for TiO_2_.

TiO_2_ particles are used as a material absorbing UV radiation in sunlight [29, 30]. Therefore, it was expected that this type of nanoparticles should provide the best protection against photodegradation. Even though the effectiveness of UV absorption of TiO_2_ is very high, TiO_2_ at the same time emits photoelectrons that can be involved in the production of peroxides and other reactive oxygen species (ROS—reactive oxygen species) [31].

Also, SiO_2_ did not improve the stability of the polymer against UV irradiation. Of all the ceramic particles tested, SiO_2_ is the most transparent to UV radiation. Phase transition energies also fell the fastest in this case. Also, the presence of SiO_2_ in the fibers promoted their degradation due to the possibility of UV radiation passing through the SiO_2_ grains (present on the surface and inside the fiber). In addition, the PLA/SiO_2_ nonwoven had the lowest cold crystallization energy. This may indicate a large amount of the crystalline phase.

### Impact of UV aging on fibers chemical structure

**Fig. 7:**
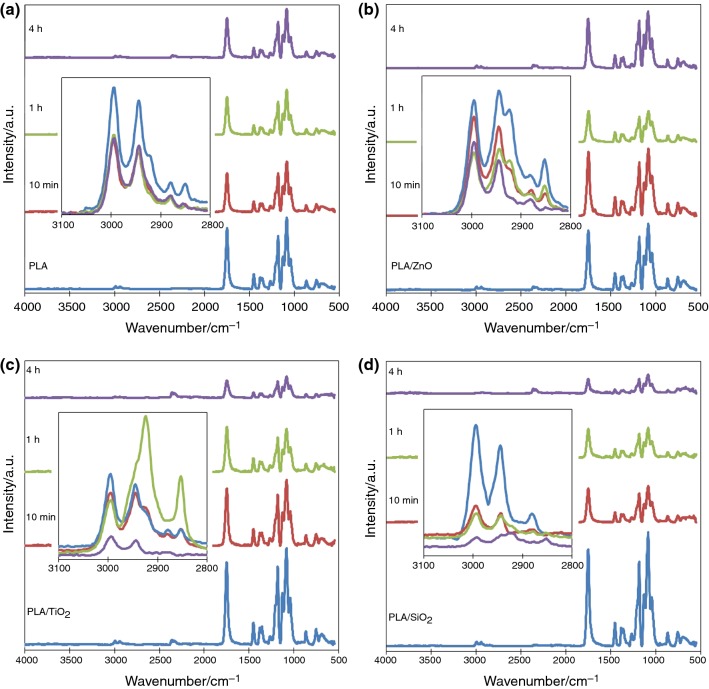
FTIR-ATR spectra of: **a** PLA fibers and composite fibers with: **b** ZnO, **c** TiO_2_, **d** SiO_2_, before and after different time of UV exposure

The effect of UV aging on the chemical structure of PLA-based fibers was examined by the FITR-ATR method. ATR spectrum of PLA nonwoven is shown in Fig. 7. Typical bands of PLA can be found in the range 3000–2800 cm^−1^ (asymmetric and symmetric stretching vibrations in CH_3_, CH_2_ and CH groups), at 1382.0 cm^−1^ (symmetric bending vibration of C–H) and 866.1 cm^−1^ (stretching vibration of C–C). Another characteristic peak at 1750.8 cm^−1^ corresponded to C=O groups [32]. Bands at 1181.3 cm^−1^ and 1084.4 cm^−1^ can be assigned to C–O–C vibrations of ester groups [33]. After aging changes in spectra weren’t significant. There was no appearance of new peaks as the result of polymer degradation. Only decrease in some band intensity was observed (Fig. 7a).

The spectra of different composite nonwovens looked similarly to this made of pure PLA (Fig. 7b–d). The main bands of ceramic fillers could be overlapped and covered by the bands from the polymeric matrix. In addition, method (ATR) chosen to study the effect of photodegradation on composites chemical structure measures only a thin layer having direct contact with the crystal. The infrared analysis of PLA after UV aging test did not show any significant changes of the spectrum or formation of new bands. The characteristic bands of the PLA decreased their intensities, due to the photodegradation of the polymer structure (smaller graphs show peaks for C–H stretching vibrations in PLA chains). In the case of pure PLA fibers, the intensity of bands in the range 3000–2800 cm^−1^ clearly decreased after just 10 min of UV exposure. However, an intensity of whole spectra did not change in a significant way even after 4 h (Fig. 7a). For composites with ZnO, an intensity of the C–H stretching vibrations decreased more gradually over time. After 10 min, the change seems to be less rapid than in the case of pure PLA (Fig. 7b). Again, the decrease in peaks in this area was also observed for PLA composites with TiO_2_ and SiO_2_. However, in these cases, the changes are much more significant, and after 4 h of UV exposure, the bands almost completely disappeared. Polymer degradation is also indicated by gradual disappearance of peaks in the whole range of spectra (Fig. 7c, d).

The UV absorbers in the form of ceramic nanoparticles were added to slow down the UV degradation of poly(lactic acid). Only ZnO protected the polymer matrix against photodegradation.

## Conclusions

The nanocomposite fibers based on poly(lactic acid) were produced successfully by an electrospinning method. The parameters of process (concentration of polymers, gap between needle and collector, ratio of solutions) were optimized to obtain defect-free microfibers. It turned out that the chosen method is very sensitive to changing process conditions. The diameter of the fibers can be easily controlled by increasing the distance between the needle and the collector. The longer solvent evaporation time results in smaller diameter fibers. The addition well dispersed ceramic powders to PLA-based solution completely changed nature of the produced fibers. Ceramic materials, known as UV absorbent, were incorporated into polymer matrix to slow down the aging process. Stability of PLA is a key aspect for using this biopolymer as packaging material and in tissue engineering. The protective effect was noticed only for ZnO particles. The addition of ZnO significantly increased the proportion of crystalline and amorphous phases in the fabricated PLA-based fibers and changed the mechanism of the photodegradation. Similar impact was noticed in the case of TiO_2_ and SiO_2_. These additives significantly changed the microstructure of the nonwovens and accelerated matrix degradation. They caused cracking and breaking of fibers.
